# Lymph‐Directed Self‐Immolative Nitric Oxide Prodrug for Inhibition of Intractable Metastatic Cancer

**DOI:** 10.1002/advs.202101935

**Published:** 2022-01-05

**Authors:** Taejeong Kim, Jeeyeon Suh, Jihoon Kim, Won Jong Kim

**Affiliations:** ^1^ Department of Chemistry Pohang University of Science and Technology (POSTECH) 77 Cheongam‐ro, Nam‐gu Pohang 37673 Republic of Korea; ^2^ Parker H. Petit Institute for Bioengineering and Bioscience Georgia Institute of Technology 315 Ferst Dr NW Atlanta GA 30332 USA; ^3^ OmniaMed Co. Ltd Pohang 37666 Republic of Korea

**Keywords:** lymph‐directed drug delivery, metastatic cancer therapy, nitric oxide, prodrug, redox chemistry

## Abstract

There has been a significant clinical demand for lymph‐directed anti‐metastatic therapy as tumor‐draining lymph nodes play pivotal roles in cancer metastasis which accounts for more than 90% of tumor‐related deaths. Despite the high potential of nitric oxide (NO) in anti‐cancer therapy owing to its biocompatibility and tumor cell‐specific cytotoxicity, the poor stability and lack of target specificity of present NO donors and delivery systems have limited its clinical applications. Herein, a redox‐triggered self‐immolative NO prodrug that can be readily conjugated to various materials containing free thiol groups such as albumin, is reported. The prodrug and its conjugates demonstrate smart release of NO donor via intramolecular cyclization under reductive conditions, followed by spontaneously generating NO in physiological conditions. The albumin‐prodrug conjugate inhibits tumor metastasis by inducing cytotoxicity preferentially on tumor cells after efficiently draining into lymph nodes. This novel prodrug can contribute to the development of on‐demand NO delivery systems for anti‐metastatic therapy and other treatments.

## Graphical abstract

Lymphatic delivery of SISIN‐1 conjugated albumin (AL‐SISIN‐1) for anti‐metastatic cancer therapy. Subcutaneously injected AL‐SISIN‐1 can be drained preferentially into the lymph node to release NO via self‐immolative process under reductive conditions, which exerts tumor cell‐specific cytotoxic effects.

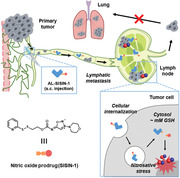

## 1 Introduction

Metastasis accounts for more than 90% of cancer deaths, which occurs due to the spread of cancer cells from their first occurrence sites to distant locations in the body.^[^ ^1^ ^]^ A lymph node is a secondary lymphoid organ that not only governs immune responses but is also involved in the clearance and filtering of damaged cells. In particular, tumor‐draining lymph nodes (TDLNs) that lie downstream of the tumor act as the primary bridgehead for the metastatic spread of cancer cells.^[^ ^2^, ^3^ ^]^ Therefore, the removal of TDLNs through surgical dissection is considered the most effective way to prevent metastasis, even in early‐stage cancer, and to treat cancer in advanced stages. In addition, various anti‐cancer drugs have been exploited to kill cancer cells in the TDLNs by utilizing passive lymphatic transport, which allows infused materials characterized by neutral or slightly negatively charged 10–30 nm flexible organic materials to be efficiently directed toward draining lymph nodes via the pressure gradient of interstitial flow.^[^ ^4^, ^5^ ^]^ However, both strategies can cause severe long‐term side effects, including a permanent breakage of the lymphatic structure, and a compromised and malfunctioning immune system, which increase the risk of lymphedema, infection, cancer reoccurrence, and autoimmune disease.^[^ ^6^ ^]^ Accordingly, there has been a significant clinical demand for noninvasive, TDLN‐targeting, and cancer cell‐specific anti‐metastatic therapy to improve the prognosis and survival rates of patients after surgical dissections of tumors, anti‐cancer drug administration, and radiation therapy.

Nitric oxide (NO) is an endogenous, gaseous, and free‐radical molecule that significantly affects various biological and physiological pathways, such as vasodilation, wound healing, anti‐inflammation, and tumor progression.^[^ ^7^, ^8^ ^]^ Therefore, several types of NO donors have been identified and developed to treat and control various diseases by artificially modulating micro‐environmental concentrations of NO at target sites.^[^ ^9^, ^10^ ^]^ In particular, NO donors have recently been highlighted in the field of anti‐cancer therapy because they can exert tumor cell‐specific cytotoxicity owing to the differences in reactive nitrogen species (RNS) capacity and sensitivity between cancer cells and normal cells. NO donors can also mitigate systemic side effects owing to their rapid decomposition into nontoxic ions abundant in the body after the completion of their local action, in contrast to conventional anti‐cancer drugs.^[^ ^11^ ^]^ In this context, we expect that the lymph‐directed delivery of NO donors can achieve efficient and safe TDLN‐targeted anti‐metastatic cancer therapy. However, the majority of NO donors exhibit poor stability, nontargeted NO‐releasing behavior, and resistance to further chemical modification.^[^ ^12^, ^13^ ^]^ These characteristics are major obstacles for the development of on‐demand NO delivery systems to deliver optimal amounts of NO to sites of interest.

**Figure 1:**
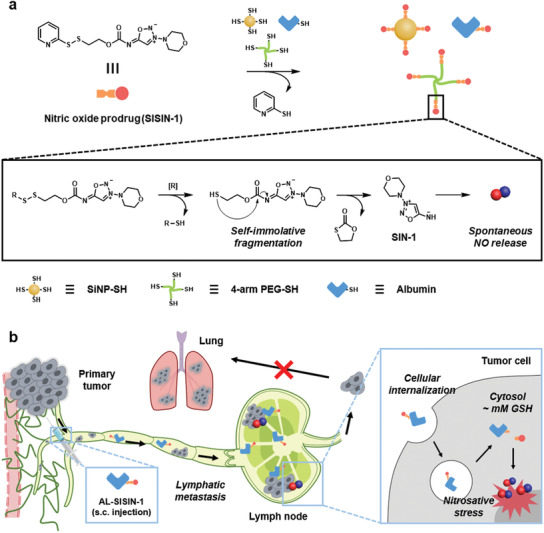
Redox‐triggered self‐immolative NO prodrug and delivery into lymph node for anti‐metastasis. a) Schematic illustration and chemical structure of the redox‐triggered self‐immolative NO prodrug (SISIN‐1). SISIN‐1 is conjugated onto various thiolated materials, which disintegrate to release a NO donor (SIN‐1) under reductive conditions. The released SIN‐1 spontaneously donates NO in physiological conditions. [R] represents the reduction. b) Lymphatic delivery of SISIN‐1 conjugated albumin (AL‐SISIN‐1) for anti‐metastatic cancer therapy. Subcutaneously injected AL‐SISIN‐1 is drained preferentially into the lymph node to release NO under reductive conditions, which exerts tumor cell‐specific cytotoxic effects.

Herein, we report an anti‐metastatic system to efficiently deliver NO to cancer cells that have spread into the TDLNs. A novel NO donor, *N*‐((2‐pyridin‐2‐yldisulfanyl)ethoxyl)carbonyl‐3‐morpholinosydnonimine (SISIN‐1), was rationally designed from 3‐morpholinosydnonimine hydrochloride (SIN‐1), not only to exhibit programmed self‐immolative fragmentation and release SIN‐1 under cytosol‐specific redox potentials, but also to be readily conjugated to various thiol‐functionalized materials, including inorganic particles, polymers, and proteins under mild synthetic conditions (**Figure** **1**a). Albumin was selected for the efficient lymphatic delivery of SISIN‐1 because of its intrinsic ability to enhance lymphatic drainage and its preferential cancer cell uptake.^[^ ^14^ ^]^ SISIN‐1 was easily conjugated to albumin (AL‐SISIN‐1), which facilitated enhanced lymphatic drainage, remarkably inhibited tumor metastasis, and prolonged the survival of mice by causing tumor cell‐specific cytotoxicity in the TDLNs (Figure 1b). Not only does AL‐SISIN‐1 provide a potential strategy to combat the metastasis of tumors but SISIN‐1 also facilitates the further development of on‐demand NO delivery systems for the treatment of various diseases.

## 2 Results and Discussion

### 2.1 Synthesis and Characterizations of Self‐Immolative Redox‐Responsive NO Prodrug (SISIN‐1)

Initially, the SISIN‐1 prodrug was rationally designed to achieve redox‐triggered self‐immolative fragmentation and facile chemical functionalization (Figure 1a). In detail, a disulfide linkage that can be readily cleaved under cytosolic reductive conditions was introduced, of which end contained 2‐pyridyl disulfide and the other end was conjugated with SIN‐1, an NO donor. A free thiol reduced from a disulfide linkage initiated the intramolecular nucleophilic attack and cleaved the carbamate linked with SIN‐1 via the formation of a five‐membered cyclic ring, thereby allowing the release of free SIN‐1. Free SIN‐1 released from SISIN‐1 spontaneously decomposed to release NO under physiological conditions. In addition, the 2‐pyridyl disulfide moiety was designed to function as an excellent cleaving group for facile integration into various thiol‐functionalized materials via a thiol‐exchange reaction. To the best of our knowledge, this is the first NO donor to exploit a self‐immolative function.

**Figure 2:**
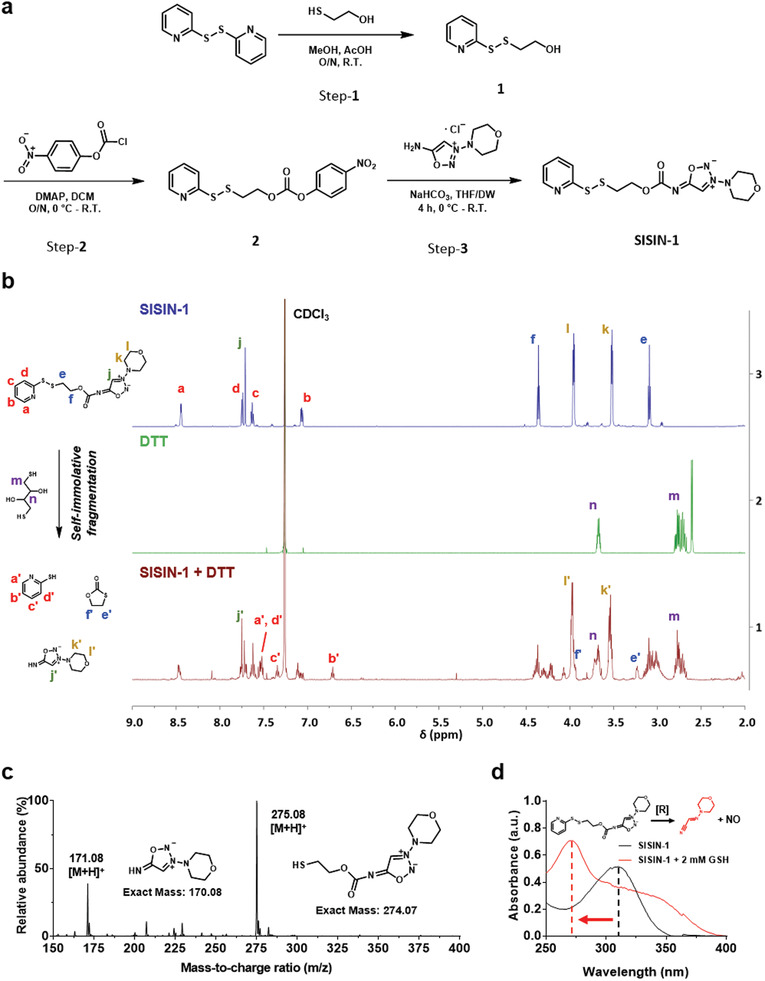
Preparation of SISIN‐1 and its self‐immolative disintegration under reductive conditions. a) Synthetic route of self‐immolative NO prodrug (SISIN‐1) preparation. b) ^1^H NMR spectra for redox‐triggered self‐immolative fragmentation of SISIN‐1. 2 equivalence of dithiothreitol (DTT, reducing agent) was treated into SISIN‐1 in CDCl_3_ and incubated at room temperature overnight. c) Mass spectra (ESI^+^) of redox‐triggered self‐immolative fragmentation for SISIN‐1. 5 equivalence of dithiothreitol (DTT, reducing agent) was treated into SISIN‐1 in acetonitrile and incubated for 2 h at room temperature to monitor the reaction. Major peaks at 171.08 and 275.08 *m*/*z* represent the whole molecule of each product. d) UV/vis absorbance spectra of SISIN‐1 after the treatment of 2 mm GSH. SISIN‐1 was incubated with 2 mm GSH in 5% DMSO in distilled water at 37 °C for 2 h before analysis.

The designed SISIN‐1 prodrug was prepared by stepwise synthesis from 2,2′‐dithiodipyridine (**Figure** **2**a). In brief, 4‐nitrophenyl chloroformate was conjugated with a hydroxyl group in the presence of 4‐dimethylaminopyridine (DMAP) as a base after substituting the 2‐mercaptopyridine moiety with 2‐mercaptoethanol. SISIN‐1 was then synthesized by nucleophilic substitution of SIN‐1, forming a carbamate group under slightly basic conditions. The successful synthesis of SISIN‐1 was confirmed by ^1^H‐nuclear magnetic resonance (^1^H NMR) spectroscopy (Figure S1, Supporting Information).

The redox‐triggered self‐immolative fragmentation of the SISIN‐1 prodrug was demonstrated by confirming the degradants in the ^1^H NMR spectra and the electrospray ionization (ESI^+^) mass spectra. The characteristic peaks for the 2‐mercaptopyridine moiety, 1,3‐oxathiolan‐2‐one, and SIN‐1 were all observed in the ^1^H NMR spectra only under a reductive condition (2 equiv. dithiothreitol (DTT)), which are consistent with the predicted intramolecular cyclization mechanism (Figure 2b; Figures S2 and S3, Supporting Information). In addition, treatment with DTT for 2 h reduced the disulfide linkage of SISIN‐1, generating two main peaks corresponding to SIN‐1 and free thiol‐containing SISIN‐1 in the ESI^+^ mass spectra (Figure 2c). In the ultraviolet/visible (UV/vis) absorbance spectra, the characteristic absorption peak of SISIN‐1 at 310 nm, corresponding to the heterocyclic sydnone imine group from SIN‐1, shifted to 270 nm at intracellular concentrations of glutathione (GSH, 2 mm), but not under extracellular concentrations (20 µm) (Figure 2d). These results indicate the spontaneous breakage of the N—N bond within the sydnone imine moiety after self‐immolative fragmentation of the SISIN‐1 prodrug. The amount of NO released by SISIN‐1 was significantly higher at intracellular GSH concentrations (2 mm) than at extracellular concentrations (20 µm) (Figure S4, Supporting Information), suggesting a dependence of SISIN‐1 degradation on the redox potential.

### 2.2 Facile Integrations of SISIN‐1

The facile functionalization of various thiol‐functionalized materials with SISIN‐1 was investigated. Thiolated silica nanoparticles (SiNP‐SH) were employed to explore the functionalization of inorganic materials with SISIN‐1 via a thiol‐disulfide exchange reaction (Figure S5a, Supporting Information). The presence of nitrogen atoms in the energy‐dispersive X‐ray spectroscopy mapping images (Figure S5b,c, Supporting Information) and the characteristic peaks for SISIN‐1 at 1580 and 1670 cm^−1^ (C═N stretching and C═O stretching, respectively) in the Fourier‐transform infrared (FT‐IR) spectra (Figure S5d, Supporting Information) demonstrated the successful conjugation of SISIN‐1 onto SiNP‐SH. Thermogravimetric analysis revealed that SISIN‐1 accounted for 3.2 wt% of the resultant SISIN‐1‐conjugated SiNPs (SiNP‐SISIN‐1) (Figure S5e, Supporting Information). In line with free SISIN‐1, SiNP‐SISIN‐1 released a significantly larger amount of NO at intracellular redox concentrations ([GSH] = 2 mm) than in the extracellular environment ([GSH] = 20 µm) (Figure S5f, Supporting Information). Four‐arm thiolated polyethylene glycol (PEG‐SH) was selected as a representative thiolated polymer to demonstrate the facile conjugation of SISIN‐1 (Figure S6a, Supporting Information). A four‐arm PEG‐SISIN‐1 was synthesized by conjugating SISIN‐1 onto four‐arm PEG‐SH under aqueous conditions. The resultant four‐arm PEG‐SISIN‐1 contained ≈1.8 SISIN‐1 moieties per polymer, which was confirmed by the presence of morpholine in the ^1^H NMR spectra (Figure S6b, Supporting Information), as well as an increase in molecular weight in the matrix‐assisted laser desorption/ionization‐time of flight (MALDI‐TOF) mass spectra and in the gel permeation chromatography (GPC) profile (Figure S7a,b, Supporting Information). Further confirmation was provided by the characteristic peaks for SISIN‐1 in the FT‐IR spectra (Figure S7c, Supporting Information). The four‐arm PEG‐SISIN‐1 also exhibited intracellular GSH‐responsive NO‐release behavior (Figure S7d, Supporting Information). These results suggest the facile functionalization of SISIN‐1 onto any type of biomaterial functionalized with thiol groups, which further raises the expectation of the development of on‐demand and controlled NO delivery systems, followed by their practical application in the treatment of various diseases.

### 2.3 Development of an Albumin‐Based SISIN‐1 Delivery System (AL‐SISIN‐1)

A lymph‐directed delivery platform is required to efficiently deliver the developed SISIN‐1 to the TDLNs.^[^ ^15^, ^16^, ^17^ ^]^ Considering the biocompatibility and effective hydrodynamic diameter of macromolecular structures for passive lymphatic drainage (≈10 nm),^[^ ^4^, ^18^ ^]^ albumin is one of the most widely employed platforms for developing lymphatic drug delivery systems.^[^ ^5^, ^19^, ^20^ ^]^ Indeed, subcutaneously administered fluorescein isothiocyanate‐labeled bovine serum albumin (BSA‐FITC, ≈10 nm) displayed significantly higher fluorescence in the homogenates of lymph nodes than small molecular free fluorescein (Figures S8 and S9, Supporting Information), demonstrating the efficient lymphatic uptake of albumin. In addition, albumin has been reported to possess an active tumor‐targeting capability through gp60‐ and secreted protein, acidic and rich in cysteine (SPARC) receptor‐mediated transcytosis in the context of anti‐metastatic cancer therapy.^[^ ^14^ ^]^ Accordingly, we designed AL‐SISIN‐1 to achieve the efficient lymphatic delivery of NO, particularly for the treatment of lymphatic cancer metastasis.

**Figure 3:**
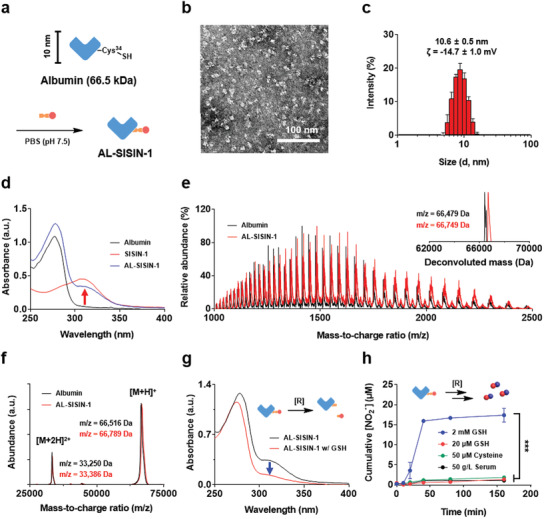
Characterization of AL‐SISIN‐1. a) Preparation scheme of AL‐SISIN‐1 in physiological conditions. b) Representative TEM image of AL‐SISIN‐1. c) Average hydrodynamic size and surface charge (*ζ*) of AL‐SISIN‐1 determined by dynamic light scattering (DLS). d) UV/vis absorbance spectra of albumin, free SISIN‐1, and AL‐SISIN‐1. Red arrow indicates the characteristic absorbance of SISIN‐1 (310 nm). e) High‐resolution mass spectra (ESI^+^) of albumin and AL‐SISIN‐1 obtained by a quadrupole time‐of‐flight mass spectrometer (Q‐TOF‐MS). Each sample was sprayed with 0.1% trifluoroacetic acid (pH 2). Inset image represents the corresponding deconvoluted mass spectra. f) MALDI‐TOF mass spectra of albumin and AL‐SISIN‐1. g) UV/vis absorbance spectra of AL‐SISIN‐1 2 h after treatment of GSH at 37 °C. h) Griess assay‐assisted cumulative NO release profile of AL‐SISIN‐1 (50 µm) under extracellular serum (50 g L^−1^ serum), physiological cysteine concentration (50 µm), and extracellular (20 µm GSH) and intracellular (2 mm GSH) reductive conditions at 37 °C in Dulbecco's modified Eagle's medium. Data are presented as mean ± SD (*n* = 4), which were statistically analyzed using two‐way ANOVA. ***p < 0.001.

SISIN‐1 was modified onto the Cys‐34 residue of bovine serum albumin under physiological conditions (phosphate‐buffered saline (PBS), pH 7.5) (**Figure** **3**a). Of note, the bovine serum albumin contains only one free thiol group at Cys‐34 residue to involve in a thiol‐disulfide exchange reaction. The resulting AL‐SISIN‐1 with a size of 10.6 ± 0.5 nm was uniformly distributed (Figure 3b and 3c). It exhibited characteristic absorption peaks at 280 and 310 nm corresponding to the Trp‐Tyr residues from albumin and the heterocyclic sydnone imine group from SIN‐1, respectively (Figure 3d). The successful synthesis of AL‐SISIN‐1 was assessed using ESI^+^ mass spectroscopy and MALDI‐TOF mass spectroscopy (Figure 3e,f). Notably, the mass‐to‐charge ratio (*m*/*z*) increased after SISIN‐1 conjugation (273 g mol^−1^), which agreed with the theoretical mass of the conjugated form of SISIN‐1 (272.3 *m*/*z*), indicating a one‐to‐one reaction between SISIN‐1 and Cys‐34 residue of albumin. The decrease in the intensity of the characteristic peak (310 nm) at an intracellular reductive concentration ([GSH] = 2 mm) in the UV/vis absorbance spectra demonstrated the intracellular redox‐responsive behavior of AL‐SISIN‐1 (Figure 3g). In line with free SISIN‐1, four‐arm PEG‐SISIN‐1, and SiNP‐SISIN‐1, AL‐SISIN‐1 released a significant amount of NO at an intracellular redox condition ([GSH] = 2 mm) (Figure 3h). In contrast, negligible NO release was observed in the presence of serum (50 g L^−1^), physiological cysteine concentrations (50 µm), or at extracellular redox conditions ([GSH] = 20 µm), which represents the extracellular redox potentials of systemic tissues, including blood and skin.^[^ ^21^ ^]^ These results imply that AL‐SISIN‐1 exhibits both adequate stability during circulation and selective intracellular NO delivery.

### 2.4 Efficient Lymphatic Delivery of AL‐SISIN‐1

**Figure 4:**
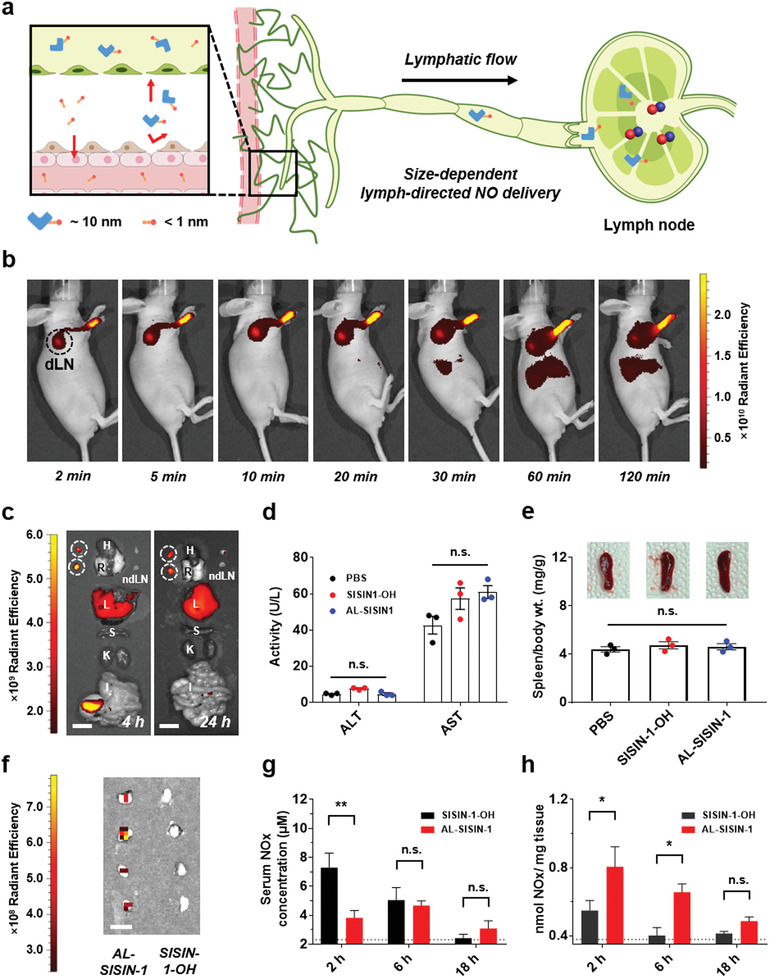
Lymphatic drainage and accumulation of AL‐SISIN‐1. a) Schematic illustration of size‐dependent lymphatic drainage and accumulation of SISIN‐1 prodrug. Hydrodynamically larger AL‐SISIN‐1 (≈10 nm) allows the enhanced lymphatic uptake relative to small molecules (<1 nm). b) IVIS fluorescence image of time‐dependent lymphatic accumulation and biodistribution for AF647‐labeled AL‐SISIN‐1 (AF647‐AL‐SISIN‐1). AF647‐AL‐SISIN‐1 (2 mm, 10 µL) was visualized by intravital fluorescence imaging (excitation/emission peak at 640/710 nm). White dot circles represent the draining lymph nodes. c) Ex vivo fluorescence image of major organs including lymph node, heart, lung, liver, spleen, kidney, and intestine 4 and 24 h after the sample treatment of AF647‐AL‐SISIN‐1. White dot circles represent the draining lymph nodes. H, R, ndLN, L, S, K, and I represent heart, lung, non‐draining lymph node, liver, spleen, kidney, and intestine, respectively. Scale bar is 1 cm. d) ALT/AST analysis for blood samples 3 days after the sample treatment. Data are presented as mean ± SEM (*n* = 3), which were statistically analyzed using one‐way ANOVA; n.s. represents no significant difference. e) Quantification of the corresponding spleen/body weight ratio for investigation of systemic toxicity of AL‐SISIN‐1. Spleens were collected 3 days after the treatment of samples. Inset images are the representative photograph of spleens collected from 8‐week old female BALB/c 3 days after treatment of samples. Data are presented as mean ± SEM (*n* = 3), which were statistically analyzed with one‐way ANOVA. n.s. represents no significant difference. f) Ex vivo fluorescence image of size‐dependent lymphatic drainage for NO donation. 2 h after the subcutaneous sample injection (2 mm, 10 µL), the draining lymph nodes were collected to assess the amount of NO delivered by AL‐SISIN‐1. 1:1 solution of DAF‐2 DA (5 µm) and 1% mercury(II) chloride was injected for imaging. Scale bar is 0.5 cm. g,h) Nitrite/Nitrate Assay Kit‐assisted quantification of NO_x_ levels in g) serum and h) draining lymph nodes after the sample treatment (2 mm, 30 µL) subcutaneously into the paw of 8‐week old female BALB/c mice. Dashed line represents the NO_x_ level from the saline‐treated group of mice. Data are presented as mean ± SEM (*n* = 3), which were statistically analyzed using two‐way ANOVA. *p < 0.05, **p < 0.01; n.s. represents no significant difference.

The size of AL‐SISIN‐1 (10.6 ± 0.5 nm) implied that it could achieve efficient NO delivery to lymph nodes via passive lymphatic transport (**Figure** **4**a). Considering the potential chemical reaction of SISIN‐1 with various thiol‐containing endogenous biomolecules, a small molecular alcohol‐conjugated SISIN‐1 (SISIN‐1‐OH, <1 nm) was prepared as a control substance via a thiol‐disulfide exchange reaction between SISIN‐1 and 2‐mercaptoethanol (Figures S10 and S11, Supporting Information). The time‐dependent lymphatic drainage and biodistribution of AL‐SISIN‐1 were tracked via an in vivo imaging system (IVIS) using AF647‐labeled AL‐SISIN‐1 (AF647‐AL‐SISIN‐1). Subcutaneously administered AF647‐AL‐SISIN‐1 started to drain into and accumulate in the lymph nodes within 2 min and continued for over 24 h, which was attributed to the passive lymphatic transport of albumin (Figure 4b,c; Figures S12 and S13, Supporting Information). The accumulation of AL‐SISIN‐1 in the liver was attributed to the intrinsic metabolic clearance of albumin.^[^ ^22^ ^]^ Nevertheless, the levels of alanine aminotransferase (ALT) and aspartate aminotransferase (AST) activity, indicative of systemic liver toxicity, did not change in the blood (Figure 4d). A negligible hemoglobin release was observed in the blood serum in the hemolysis assay, suggesting good blood compatibility of AL‐SISIN‐1 (Figure S14, Supporting Information). Moreover, inflammation‐associated spleen enlargement was not detected 3 days after sample treatment (Figure 4e). In hematoxylin and eosin (H&E) histological assays, no significant morphological differences were observed in the histology of major organ tissues, including the heart, lungs, kidney, liver, spleen, and draining lymph nodes 3 days after treatment with AL‐SISIN‐1 (Figure S15, Supporting Information). These results demonstrated that AL‐SISIN‐1 induced negligible systemic toxicity despite systemic exposure after subcutaneous administration. Lymphatic NO delivery was also visualized using a fluorescent NO probe (diaminofluorescein‐2 diacetate, DAF‐2DA) 2 h after subcutaneous administration of AL‐SISIN‐1. As expected, the macromolecular AL‐SISIN‐1 produced stronger fluorescence signals in draining lymph nodes than small molecular SISIN‐1‐OH (Figure 4f). In particular, AL‐SISIN‐1 donated a significantly larger amount of NO_x_ to the lymph nodes than SISIN‐1‐OH without releasing NO_x_ in bursts (Figure 4g,h), demonstrating the efficient lymphatic NO‐delivery ability of AL‐SISIN‐1.

### 2.5 Preferential Cytotoxicity of AL‐SISIN‐1 on Cancer Cells

**Figure 5:**
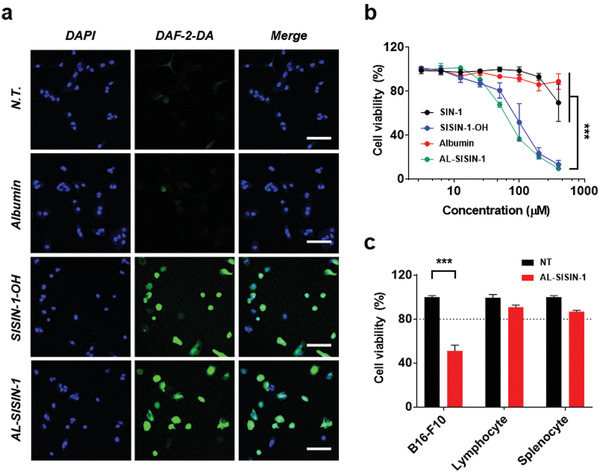
Cytosol‐selective NO release for cancer cell‐specific cytotoxic effects in vitro. a) Confocal microscopy images of B16‐F10 metastatic cancer cell lines after albumin, SISIN‐1‐OH, and AL‐SISIN‐1 (50 µm) treatments. Nuclei and intracellular NO were co‐stained with DAPI (blue) and DAF‐2 DA (green), respectively. Scale bar is 100 µm. b) Dose‐dependent anti‐cancer effects of free SIN‐1, SISIN‐1‐OH, albumin, and AL‐SISIN‐1 in B16‐F10. Relative cell viability (%) was determined 48 h post‐treatment by MTT assay. Data are presented as mean ± SD (*n* = 6), which were statistically analyzed using two‐way ANOVA. ***p < 0.001. c) Relative in vitro cell viability (%) of B16‐F10, lymphocytes, and splenocytes 48 h after treatment of AL‐SISIN‐1 (50 µm) by MTS assay. NT represents nontreatment. Data are presented as mean ± SD (*n* = 6), which were statistically analyzed using Student's *t*‐test. ***p < 0.001; n.s. represents no significant difference.

The intracellular delivery of exogenous NO has been widely reported to exert tumor cell‐specific cytotoxicity.^[^ ^11^ ^]^ We expected that lymphatic NO delivery with AL‐SISIN‐1 would inhibit tumor metastasis. First, the suitability of AL‐SISIN‐1 for intracellular delivery of NO was investigated in vitro by evaluating the fluorescent signals of DAF‐2 DA in the metastatic B16‐F10 melanoma cell line. Albumin is expected to be actively uptaken by Ras, gp60, and SPARC‐overexpressing B16‐F10 cells via endocytosis, macropinocytosis, and g60‐ and SPARC receptor‐mediated transcytosis although the exact mechanism is not yet entirely elucidated.^[^ ^23^, ^24^, ^25^ ^]^ High fluorescence intensity was observed in B16‐F10 cells after treatment with either AL‐SISIN‐1 or SISIN‐1‐OH, indicating the efficient cytosolic delivery of NO owing to their intracellular GSH‐responsive behaviors (**Figure** **5**a). Moreover, SISIN‐1‐OH and AL‐SISIN‐1 exerted significantly higher cytotoxicity on B16‐F10 metastatic cancer cells than a similar dose of free SIN‐1 (Figure 5b). The negligible cytotoxicity of free SIN‐1 was attributed to spontaneous decomposition in the medium, implying that site‐specific intracellular NO release is critical for anti‐cancer effects. More importantly, AL‐SISIN‐1 exerted insignificant cytotoxicity (cellular viability >80%) in non‐targeted cells, including lymphocytes and splenocytes (Figure 5c), suggesting the cancer cell‐specific cytotoxicity of AL‐SISIN‐1. These results highlight the potential of AL‐SISIN‐1 for the inhibition of lymphatic metastasis by inducing cytotoxicity preferentially in cancer cells after efficient lymphatic delivery.

### 2.6 In Vivo Anti‐Metastatic Effects of AL‐SISIN‐1

**Figure 6:**
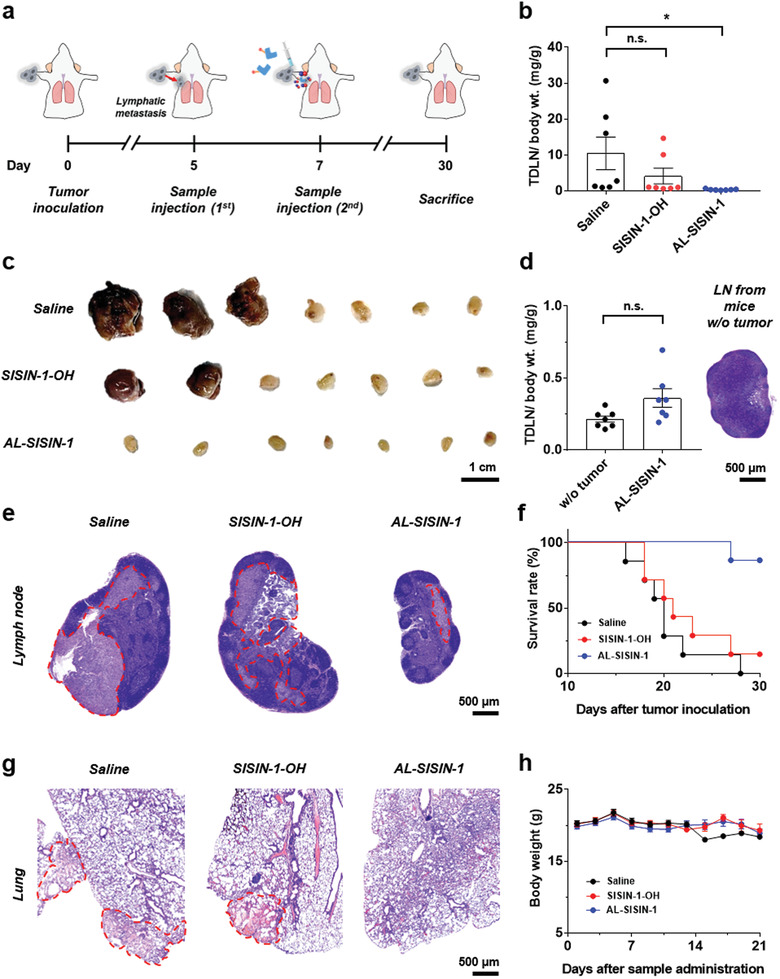
Anti‐metastatic cancer therapy with lymphatic delivery of AL‐SISIN‐1 in vivo. a) Overall experimental timeline for the in vivo experiment. 5 days after the inoculation of the B16‐F10 metastatic cancer cell lines, samples (2 mm, 10 µL) were administered subcutaneously into the paws of 8‐week‐old female BALB/c‐*nu*/*nu* mice twice. Saline and SISIN‐1‐OH were used as control groups. b) TDLN to body weight ratio to quantify the lymph node enlargement. Data are presented as mean ± SEM (*n* = 7), which were statistically analyzed using Student's *t*‐test. *p < 0.05; n.s. represents no significant difference. c) Photograph of the TDLNs excised from B16‐F10 tumor‐bearing BALB/c‐*nu*/*nu* mice after the sample treatment. d) Comparison of TDLN to body weight ratio of AL‐SISIN‐1 treated mice with that of naive mice. Data are presented as mean ± SEM (*n* = 7), which were statistically analyzed using Student's *t*‐test. n.s. represents no significant difference. Inset image represents the H&E stained lymph node (LN) of naive mice. e) Histological assay with H&E staining on TDLN sections collected from mice treated with saline, SISIN‐1‐OH, and AL‐SISIN‐1. Red dot areas indicate the infiltrated metastatic tumor cells. f) Morbidity‐free survival curve of different groups of mice after the sample treatment (*n* = 7). Mice were sacrificed when the primary or tumor‐draining lymph node volume reaches 2500 mm^3^. g) Histological assay with H&E staining for lung sections collected from mice treated with saline, SISIN‐1‐OH, and AL‐SISIN‐1. Red dot areas indicate melanoma lung modules. h) Body weight measurement after the sample treatment. Data are presented as mean ± SEM (*n* = 7).

Finally, the anti‐metastatic effects of AL‐SISIN‐1 were evaluated in a lung metastasis mouse model with tumor inoculations on the forefoot pad. This model mimics metastatic tumors initiated from melanomas on the arm and hands, accounting for ≈15% of skin melanoma.^[^ ^26^ ^]^ B16‐F10 tumors were inoculated into the right forearms of BALB/c‐*nu*/*nu* mice on day 0, followed by subcutaneous treatment with AL‐SISIN‐1 to the same forearm away from the primary tumor site on days 5 and 7 (**Figure** **6**a). AL‐SISIN‐1 treatment resulted in negligible enlargement of the lymph nodes, in contrast to saline and SISIN‐1‐OH (Figure S16, Supporting Information). To demonstrate that the inhibition of lymph node enlargement can be attributed to the prevention of lymphatic metastasis, TDLNs were excised and analyzed when the mice had died or had been sacrificed. AL‐SISIN‐1 significantly reduced the volume of TDLNs (Figure 6b,c), whereas the volume of TDLNs treated with SISIN‐1‐OH was statistically similar to that of TDLNs treated with saline (Figure 6d). In particular, histological analysis revealed that numerous infiltrated metastatic tumor cells were found in the enlarged TDLNs from saline‐ or SISIN‐1‐OH‐treated mice (Figure 6e). Conversely, the TDLNs of the mice treated with AL‐SISIN‐1 contained much fewer metastatic tumor cells than the control groups. Moreover, the effects of AL‐SISIN‐1 on anti‐lymphatic metastasis resulted in prolonged survival times compared to saline or SISIN‐1‐OH treatment (Figure 6f).

Metastatic cancer cells in TDLNs usually induce fatal pulmonary metastasis by spreading to the lung tissues, resulting in an increase in the acute death risk of cancer patients by dyspnea.^[^ ^27^, ^28^ ^]^ To determine whether the suppression of lymphatic metastasis by AL‐SISIN‐1 leads to the inhibition of pulmonary metastasis, tumor nodules from lung tissue were assessed via H&E staining. In contrast to the control groups, neither lung tumor nodule formation (Figure 6g) nor significant body weight changes (Figure 6h) were observed in AL‐SISIN‐1‐treated mice. These results demonstrate that AL‐SISIN‐1 not only inhibits lymphatic metastasis but also prevents secondary metastasis initiated by the TDLNs.

## 3 Conclusion

In conclusion, we successfully designed and synthesized a novel NO prodrug (SISIN‐1) to release NO via redox‐triggered self‐immolative fragmentation. As demonstrated with materials containing SiNP, four‐arm‐PEG, and albumin, SISIN‐1 was readily conjugated onto various materials functionalized with thiol groups, raising the possibility of on‐demand and controlled NO delivery. In particular, nanometer‐sized, SISIN‐1‐conjugated albumin exhibited enhanced lymphatic drainage and the cytosol‐selective release of NO. This suppressed lymphatic metastasis and secondary metastasis to the lungs by inducing tumor cell‐specific cytotoxic effects without significant systemic side effects. This novel prodrug shows great potential not only to provide a tool for investigating the biological pathway of NO but also to take a step forward in the clinical translation of NO‐mediated therapy including anti‐metastasis.

## 4 Experimental Section

### Reagent

All chemicals were used as provided without further purification unless indicated. Distilled water was prepared using a MilliQ filtration system (Millipore, Bedford, MA, USA). 2,2′‐dithiodipyridine, methanol, glacial acetic acid, 2‐mercaptoethanol, DCM (anhydrous), tetrahydrofuran (THF), (3‐mercaptopropyl)trimethoxysilane, ammonia solution (28–30%), BSA, dl‐dithiothreitol, 2‐mercaptopyridine, ethanolamine, dl‐cysteine hydrochloride, acetonitrile, thiazolyl blue tetrazoliumbromide (MTT), GSH, Nitrite/Nitrate Assay Kit, BSA‐FITC, fluorescein, and mercury(II) chloride were purchased from Sigma‐Aldrich Co. (St. Louis, MO, USA). DMAP and 4‐nitrophenyl chloroformate were purchased from TCI Co. (Tokyo, Japan). Ethyl acetate (EA), hexane, dichloromethane, sodium bicarbonate, and anhydrous magnesium sulfate were purchased from Samchun Chemicals (Pyeongtaek, Korea). 3‐morpholinosydnonimine hydrochloride (SIN‐1, hydrochloride) was purchased from Cayman Chemicals (Ann Arbor, MI, USA). Four‐arm PEG‐SH of which molecular weight is 10 kDa was purchased by SunBio, Inc. (Anyang, Korea). Griess assay kit was purchased from Invitrogen (Carlsbad, CA, USA). Dulbecco's phosphate‐buffered saline (DPBS), Dulbecco's modified eagle medium (DMEM), RPMI 1640 medium, 100× penicillin–streptomycin (P/S) solution (10 kU mL^−1^ penicillin and 10 mg mL^−1^ streptomycin), fetal bovine serum (FBS), and ACK lysis buffer were purchased from Capricorn Scientific (Ebsdorfergrund, Germany). DAF‐2 DA was purchased from Abcam (Cambridge, MA, USA). CellTiter 96 AQueous One Solution Cell Proliferation Assay Kit (MTS assay) was purchased from Promega Inc. (Madison, WI, USA). Albumin from BSA, Alexa Fluor 647 conjugate was purchased from Thermo Fisher Scientific (Waltham, MA, USA). Both BALB/c and BALB/c‐*nu*/*nu* mice were purchased from Orient Bio (Gyeonggi, Korea). All animal experiments were performed under the guidelines from the POSTECH Institutional Animal Care and Use Committee (IACUC) under POSTECH Biotech Center Ethics Committee.

### Instrumental Methods

^1^H NMR spectra were recorded by using Bruker Advance 500 or 600 MHz FT‐NMR. The distribution and the size of the AL‐SISIN‐1 were observed by using Zetasizer Nano (Malvern Instruments, Malvern, UK). EDS images were taken by Cs‐corrected STEM (JEM 2100F, JEOL, Tokyo, Japan). FT‐IR spectra were recorded by Vertex 70 spectrophotometer (Bruker Optics, Germany). Mass contents were determined by a thermogravimetry analyzer (TG‐2171, Cahn Instrument Inc., Cerritos, CA, USA). High‐resolution mass spectra (electrospray ionization positive mode, ESI^+^) were recorded with an Agilent 6560 ESI quadrupole time‐of‐flight (ESI‐Q‐TOF) mass spectrometer (Agilent Technologies, CA, USA) equipped with a dual AJS ESI source. MALDI‐TOF mass spectra were recorded with an Autoflex Speed LRF mass spectrometer (Bruker Daltons, Bremen, Germany). The molecular weight and polydispersity index of the block copolymer were conducted with a GPC system equipped with three linked Styragel columns (HR1, HR2, and HR4) and refractive index (RI) detector (2414 refractometer) using THF as an eluent (e2695, Waters Alliance, MA, USA). UV–vis spectra were recorded by using UV 2550 spectrophotometer (Shimadzu, Japan). The absorbance for the Griess assay, MTT, and MTS assay was determined by a multi‐mode microplate reader SpectraMax i3 (Molecular device, CA, USA). Fluorescence images of sample‐treated mice were obtained using IVIS Lumina II system (Caliper Life Sciences, MA, USA). A confocal laser scanning microscope image was obtained using Leica TCS SP5II MP SMD FLIM (Leica, IL, USA). Nikon Eclipse 80i (Tokyo, Japan) was used for the histological assay images.

### Synthesis of Self‐Immolative NO Prodrug (SISIN‐1)

The synthesis of self‐immolative NO prodrug (SISIN‐1, *N*‐((2‐pyridin‐2‐yldisulfanyl)ethoxyl)carbonyl‐3‐morpholinosydnonimine) was carried out in three steps and all reaction products were confirmed by ^1^H NMR.

Step‐1 (synthesis of compound **1**): Briefly, 210 µL of glacial acetic acid was added to the 2,2′‐dithiodipyridine (4000 mg, 18.16 mmol) dissolved in 50 mL methanol. To the stirred solution, 2‐mercaptoethanol (848.9 µL, 12.10 mmol) was added dropwise and then the reaction mixture was stirred vigorously at room temperature overnight. After confirming the completion of the reaction by thin‐layer chromatography (TLC), the excess organic solvent was evaporated under reduced pressure. The resultant product, which is termed compound **1** was purified by silica gel chromatography using EA/hexane as an eluent system. The polarity of eluent was increased to 80:20 (EA/hexane) to obtain the desired product as a pale‐yellow oil (1772 mg, yield: 78.2%). ^1^H NMR (500 MHz, CDCl_3_, 25 °C, *δ*): 8.51 (ddd, *J* = 5.0, 1.7, 0.9 Hz, 1H), 7.58 (ddd, *J* = 8.0, 7.4, 1.8 Hz, 1H), 7.40 (dt, *J* = 8.1, 1.0 Hz, 1H), 7.16 (ddd, *J* = 7.4, 5.0, 1.0 Hz, 1H), 5.71 (t, *J* = 7.1 Hz, 1H), 3.81 (td, *J* = 7.0, 5.2 Hz, 2H), 3.00–2.91 (m, 2H).

Step‐2 (synthesis of compound **2**): 4‐nitrophenyl chloroformate (3.23 g, 16.0 mmol) was added to a cold (ice bath) solution of the compound **1** (1.50 g, 8.00 mmol) and DMAP (1.96 g, 16.0 mmol) in 50 mL of dry DCM. The reaction was performed under a nitrogen atmosphere overnight allowed to reach room temperature gradually. After confirming the completion of the reaction by TLC, the excess organic solvent was evaporated under reduced pressure. The resultant product, which is termed compound **2** was purified by silica gel chromatography using DCM as an eluent system to yield the desired product as a pale‐yellow liquid (2507 mg, yield: 88.8%). ^1^H NMR (500 MHz, CDCl_3_, 25 °C, *δ*): 8.51–8.48 (m, 1H), 8.31–8.26 (m, 1H), 7.70–7.62 (m, 2H), 7.41–7.36 (m, 1H), 7.12 (ddd, *J* = 6.7, 4.8, 1.8 Hz, 1H), 4.57 (t, *J* = 6.4 Hz, 1H), 3.16 (t, *J* = 6.4 Hz, 1H). z

Step‐3 (synthesis of **SISIN‐1**): 3‐morpholinosydnonimine hydrochloride (SIN‐1, 300 mg, 1.45 mmol), and sodium bicarbonate (150 mg, 1.78 mmol) were mixed in 2.5 mL of an aqueous system. The freshly prepared SIN‐1 mixture was added to compound **2** (394 mg, 1.12 mmol) dissolved in 5 mL THF, followed by vigorously stirring at room temperature for 4 h. After confirming the completion of the reaction by TLC, THF was evaporated and the remaining aqueous phase was extracted with DCM, dried, filtered, and rotary evaporated. The residue was purified by column chromatography using neutral aluminum oxide as a stationary phase and 1% methanol in DCM as an eluent system to obtain an ivory sticky liquid. The resultant product is termed **SISIN‐1** (377 mg, yield: 88.1%). ^1^H NMR (600 MHz, CDCl_3_, 25 °C, *δ*): 8.44 (d, *J* = 4.6 Hz, 1H), 7.74 (d, *J* = 8.1 Hz, 1H), 7.71 (s, 1H), 7.63 (td, *J* = 7.8, 1.7 Hz, 1H), 7.07 (dd, *J* = 7.3, 4.9 Hz, 1H), 4.39 (t, *J* = 6.6 Hz, 2H), 4.02–3.96 (m, 4H), 3.59–3.52 (m, 4H), 3.12 (t, *J* = 6.6 Hz, 2H).

### Preparation of SISIN‐1 Conjugated Silica Nanoparticle (SiNP‐SISIN‐1)

Thiolated silica nanoparticle (SiNP‐SH) to be functionalized with SISIN‐1 was prepared. Briefly, (3‐mercaptopropyl)trimethoxysilane (2.0 g) was dissolved in 100 mL of distilled water. When the solution became transparent, 3.4 mL of ammonia solution was added to the solution and then the reaction mixture was stirred vigorously at room temperature for 4 h. The resultant SiNP‐SH was collected by three times repeated centrifugation and redispersion cycles, followed by replacing the supernatants with methanol.

Finally, 1 mg mL^−1^ of SiNP‐SH and 0.1 mg mL^−1^ of SISIN‐1 were mixed in methanol with 0.2% (v/v) glacial acetic acid for conjugation. After agitating the solution for 24 h under a dark condition, the SISIN‐1 conjugated silica nanoparticle (**SiNP‐SISIN‐1**) was collected by centrifugation at 4000 rpm for 10 min with distilled water. The resultant **SiNP‐SISIN‐1** was confirmed by analyzing TEM, EDS, GPC, TGA, and Griess assay.

### Preparation of SISIN‐1‐Conjugated Four‐Arm PEG (Four‐Arm PEG‐SISIN‐1)

The preparation of SISIN‐1‐conjugated four‐arm PEG (**four‐arm PEG‐SISIN‐1**) was carried out under an aqueous condition. Briefly, SISIN‐1 (23.0 mg, 0.06 mmol) in 0.1 mL DMSO was added to four‐arm PEG‐SH (100 mg, 0.01 mmol) in 5 mL distilled water. After the addition of 5.0 µL glacial acetic acid into the solution, the reaction was performed under vigorous stirring at room temperature overnight. The resulting solution was then transferred into the dialysis membrane in which molecular cutoff was 2 kDa for purification. The **four‐arm PEG‐SISIN‐1** was obtained after consecutive 2‐day dialysis against distilled water, followed by lyophilization. The resultant **four‐arm PEG‐SISIN‐1** was confirmed by analyzing ^1^H NMR, FT‐IR, GPC, mass spectra (MALDI), and Griess assay.

### Preparation of SISIN‐1‐Conjugated Albumin (AL‐SISIN‐1)

The preparation of SISIN‐1‐conjugated albumin (**AL‐SISIN‐1**) was carried out under an aqueous condition. Briefly, BSA was dialyzed against distilled water and lyophilized before use. SISIN‐1 (4.86 mg, 12.7 µmol) in 0.1 mL DMSO was added to predialyzed albumin (648 mg, 9.76 µmol) dissolved in 15 mL phosphate buffer solution (0.1 m, pH 7.5). The reaction was performed under vigorous stirring at room temperature overnight. After confirming the completion of the reaction by UV–vis absorbance, the reaction mixture was transferred to the 30 kDa molecular weight cutoff Amicon ultra centrifugal filter and centrifuged at 25 °C for 20 min at 4000 rpm. The supernatant was then washed with distilled water thrice, collected, and lyophilized. The resultant product termed **AL‐SISIN‐1** (510 mg, yield: 78.2%) was confirmed by analyzing TEM, DLS, UV–vis absorbance, and mass spectra (ESI^+^ and MALDI).

### Preparation of Alcohol‐Conjugated SISIN‐1 (SISIN‐1‐OH)

For investigation of the size‐dependent lymph‐directed drainage, the alcohol‐conjugated SISIN‐1 (**SISIN‐1‐OH**) was synthesized as a control by conjugating SISIN‐1 with mercaptoethanol. Briefly, 1.4 µL of glacial acetic acid was added to the SISIN‐1 (30 mg, 78 µmol) dissolved in 5 mL methanol under a nitrogen atmosphere. To the stirred solution, 2‐mercaptoethanol (8.5 µL, 1.2 µmol) was added dropwise and then the reaction mixture was stirred vigorously at room temperature overnight. After confirming the completion of the reaction by TLC, the excess organic solvent was evaporated under reduced pressure and purified by silica gel chromatography using 1% methanol in DCM as an eluent system to obtain the desired product as a yellow sticky liquid. The resultant product is termed **SISIN‐1‐OH** (26.9 mg, yield: 98.3%). ^1^H NMR (500 MHz, CDCl_3_, 25 °C, *δ*): 7.72 (s, 1H), 4.37 (t, *J* = 6.7 Hz, 1H), 3.99–3.95 (m, 1H), 3.02 (t, *J* = 6.6 Hz, 1H), 2.95 (t, *J* = 6.1 Hz, 1H), 2.89 (t, *J* = 5.7 Hz, 1H).

### Measurement of GSH‐Responsive NO Release

The GSH‐responsive NO release of SISIN‐1‐OH and AL‐SISIN‐1 was quantitatively investigated by using Griess assay. 150 µL of each sample in DMEM (50 µm, 1.5 mL) w/ or w/o 20 µm or 2 mm GSH at 37 °C was transferred into 96 well‐plate at a predetermined time period and stored at −70 °C for further analysis. The cumulative amount of NO in each solution was then quantified by comparing the absorbance at 548 nm with the standard curve plotted with NaNO_2_.

### Animals

All animal experiments were performed under the guidelines from the POSTECH IACUC under POSTECH Biotech Center Ethics Committee (Number: POSTECH‐2017‐0115‐R3).

### Size‐Dependent Lymph‐Directed Drainage of NO Donors

To characterize the size‐dependent lymph‐directed NO donation over time, each NO donor (2 mm, 30 µL) was subcutaneously injected into the paw tissue of 8‐week old female BALB/c mice. After the predetermined time period (2 h, 6 h, and 18 h), the sample‐treated mice were sacrificed to collect serum and the organ tissue fluids. The serum was prepared by centrifuging the clotted blood specimen in an Amicon ultra centrifugal filter at 13000 rpm for 10 min at 4 °C. The organ tissue fluids were prepared by centrifuging the organ tissue homogenate in 200 µL of cold DPBS at 13 000 rpm for 10 min at 4 °C. The NO levels of each sample were then determined using Nitrite/Nitrate Assay Kit, according to the manufacturer's protocol.

### IVIS‐Assisted Visualization of Lymph‐Directed NO Delivery

The albumin‐mediated enhancement of lymphatic‐drainage and NO donation was visualized by in vivo imaging system (IVIS). 2 h after the subcutaneous sample injection (2 mm, 10 µL), 8‐week old female BALB/c‐*nu*/*nu* mice were sacrificed. The draining lymph nodes were collected and 5 µL of a 1:1 solution of DAF‐2 DA (5 µm) and 1% mercury(II) chloride was injected. The lymph nodes were then immediately imaged using an FITC channel.

To trace the time‐dependent lymph‐directed drainage and accumulation of AL‐SISIN‐1, SISIN‐1 was first conjugated onto BSA‐AF (BSA, Alexa Fluor 647 conjugate) under a physiological condition. Briefly, AF‐647‐labeled AL‐SISIN‐1 was prepared by reacting SISIN‐1 (0.08 mg, 0.20 µmol) with BSA‐AF (10 mg, 0.15 µmol) in 2 mL phosphate buffer solution (0.1 m, pH 7.5) at room temperature for overnight, followed by Amicon filtration and lyophilization. Filtered and lyophilized AF647‐labeled AL‐SISIN‐1 (2 mm, 10 µL) was then subcutaneously injected into the paw tissue of 8‐week old female BALB/c‐*nu*/*nu* mice. The time‐dependent lymphatic accumulation and biodistribution of AL‐SISIN‐1 were visualized by intravital fluorescence imaging (excitation/emission peak at 640/710 nm).

### Hemolysis Assay

Hemolytic compatibility was evaluated by measuring the hemoglobin released from the red blood cells. The fresh mouse blood prepared in the heparin tube was centrifuged (4 °C, 15 min, 2000 rpm), and the resultant supernatant was subsequently discarded. After diluting the blood cells tenfolds in DPBS, each sample was treated to reach the final concentration at 1 mm. The blood cells were then incubated at 37 °C for 3 h, and the resulting suspensions were centrifuged (4 °C, 15 min, 2000 rpm). The absorbance of supernatants was measured at 541 nm to quantify the hemoglobin release. DPBS and 1× lysis buffer were utilized as negative (0% hemolysis) and positive control (100% hemolysis), respectively.

### H&E Histological Assay

Major organs including heart, lung, kidney, liver, spleen, and lymph node were obtained from sample‐treated euthanized mice and subsequently incubated in 10% neutral buffered formalin (NBF) at 4 °C overnight. The tissue sections were then embedded in paraffin and sliced with a microtome. Tissue slices were stained with H&E and finally monitored by microscopy for histological assay.

### Confocal Microscopy Imaging of Intracellular NO Level

The intracellular NO level of B16‐F10 cells was detected using DAF‐2 DA. Briefly, cells were seeded on the cover glass placed in a 6‐well plate at a density of 1 × 10^5^ cells per well and incubated at 37 °C under DMEM with 10% FBS, 100 U mL^−1^ penicillin, and 100 µg mL^−1^ streptomycin. After incubation overnight, old media was replaced by fresh media containing 5 µm of DAF‐2 DA and again incubated for 40 min. After that, the cells were washed with DPBS and each sample (SISIN‐1 concentrations equivalent to 50 µm) was treated with fresh media. After 3 h incubation with samples, cells were washed with DPBS and treated with 10% NBF at room temperature under a dark condition. After 30 min, the cover glass was mounted in an antifade mounting medium with DAPI to be observed by confocal microscopy.

### Dose‐Dependent In Vitro Cellular Viability on B16‐F10

The dose‐dependent in vitro cytotoxicity was evaluated after the treatment of samples including SIN‐1, SISIN‐1‐OH, albumin, and AL‐SISIN‐1 using the MTT assay. B16‐F10 cells were seeded on a 96‐well plate at an initial density of 5 × 10^3^ cells per well and incubated at 37 °C under DMEM with 10% FBS, 100 U mL^−1^ penicillin, and 100 µg mL^−1^ streptomycin. After incubation for 24 h, old media was replaced with the samples in fresh media, and the cells were again incubated for 48 h. After that, the cells were washed and cultured with the media containing 0.5 mg mL^−1^ of MTT solution for 4 h. Finally, the media were replaced with 200 µL DMSO and the corresponding absorbance at 570 nm was measured to investigate the relative cellular viability.

### In Vitro Cell‐Specific Cytotoxicity Analysis

The cellular viability of the cultured B16‐F10 cell lines, lymphocytes, and splenocytes was evaluated after the treatment of AL‐SISIN‐1 (100 µm) using MTS cell proliferation assay. Briefly, the lymphocytes and splenocytes were isolated using a cell strainer (Falcon, New Jersey, USA) from 8‐week old female BALB/c mice. The isolated cells were centrifuged at 4 °C for 5 min at 300 g and resuspended in 1 mL ACK lysing buffer on ice. After 2 min incubation, 9 mL of RPMI 1640 medium was added and the cells were again centrifuged at 4 °C for 5 min at 300 g. The isolated cells were then seeded on a 96‐well plate at an initial density of 5 × 10^3^ cells per well and incubated at 37 °C under RPMI 1640 medium with 10% FBS, 100 U mL^−1^ penicillin, and 100 µg mL^−1^ streptomycin. After treating the sample on each, the cells were incubated for 48 h and cellular viability was evaluated with MTS cell proliferation assay, according to the manufacturer's protocol.

### Anti‐Metastatic Cancer Therapy In Vivo

B16‐F10 metastatic cancer cell lines were inoculated into the paw tissue of each 8‐week old female BALB/c‐*nu*/*nu* mice (3 × 10^6^ cells per mouse). 5 days after the inoculation, the mice were randomly divided into three groups (7 mice per group). The mice were then injected with each sample (2 mm, 10 µL) twice (day 5 and 7) subcutaneously away from the primary tumor site. The morbidity‐free survival of each group was monitored and sacrificed on day 30. Mice were sacrificed when the primary or tumor‐draining lymph node volume reaches 2500 mm^3^. TDLN and lung were removed and subsequently stained with H&E to assess the infiltration of metastatic tumor cells.

### Statistical Analysis

All results are presented as the mean ± standard deviation (SD) or mean ± standard error of the mean (SEM), which are indicated in each figure. All experiments were repeated at least three times and each condition was analyzed in more than triplicate. One‐way or two‐way ANOVA tests with post‐hoc Tukey's tests were used to obtain statistical differences for multiple comparisons unless indicated. Target sample was noted as # and then compared with control groups to be evaluated, *p < 0.05, **p < 0.01, ***p < 0.001; n.s. represents no significant difference. *P*‐values less than 0.05 were considered statistically significant. A paired Student's *t*‐test was used for individual comparisons. All statistical analyses were carried out with the Prism software package (version 7.04).

## Conflict of Interest

The authors declare no conflict of interest.

## Supporting information

Supporting Information

## Data Availability

The data that support the findings of this study are available from the corresponding author upon reasonable request.
