# Supplementary material for: Lymph‐Directed Self‐Immolative Nitric Oxide Prodrug for Inhibition of Intractable Metastatic Cancer
Source: Adv Sci (Weinh). 2022 Jan 5;9(8):2101935. doi: 10.1002/advs.202101935 (PMC8922110; doi:10.1002/advs.202101935)
Supplement: Supplementary file 1 — Supporting Information [file ADVS-9-2101935-s001.pdf]

ADVANCED SCIENCE
Open Access

Supporting Information

for *Adv. Sci.*, DOI 10.1002/advs.202101935

Lymph-Directed Self-Immolative Nitric Oxide Prodrug for Inhibition of Intractable Metastatic Cancer

Taejeong Kim, Jeeyeon Suh, Jihoon Kim and Won Jong Kim**

ADVANCED SCIENCE
Open Access

Supporting Information

for *Adv. Sci.*, DOI: 10.1002/advs.202101935

Lymph-Directed Self-Immolative Nitric Oxide Prodrug for Inhibition of Intractable Metastatic Cancer

Taejeong Kim, Jeeyeon Suh, Jihoon Kim, and Won Jong Kim**

Supporting Information

Lymph-Directed Self-Immolative Nitric Oxide Prodrug for Inhibition of Intractable Metastatic Cancer

Taejeong Kim[1], *Jeeyeon Suh*[1], *Jihoon Kim**[2], *and Won Jong Kim**[1,3]

[1] Department of Chemistry, Pohang University of Science and Technology (POSTECH), 77 Cheongam-ro, Nam-gu, Pohang, 37673, Republic of Korea

[2] Parker H. Petit Institute for Bioengineering and Bioscience, Georgia Institute of Technology, 315 Ferst Dr NW, Atlanta, Georgia 30332, USA

[3] OmniaMed Co., Ltd, Pohang 37666, Republic of Korea

E-mail: jkim3441@gatech.edu; wjkim@postech.ac.kr

Table of Contents

Materials and methods

Figure S1. ^{1}H NMR spectra of synthetic compounds including SISIN-1.

Figure S2. ^{1}H NMR spectra of 2-mercaptopyridine.

Figure S3. ^{1}H NMR spectra of SISIN-1 in the presence of a primary amine and a hydroxyl group.

Figure S4. Redox-triggered NO release by SISIN-1.

Figure S5. Characterization of SiNP-SISIN-1.

Figure S6. Preparation of 4-arm PEG-SISIN-1.

Figure S7. Characterization of 4-arm PEG-SISIN-1.

Figure S8. Characteristics of BSA-FITC.

Figure S9. Size-dependent lymphatic drainage into lymph node.

Figure S10. Preparation of self-immolative small molecule NO prodrug bearing a hydroxyl group.

Figure S11. Cumulative NO release profile of SISIN-1-OH under reductive conditions.

Figure S12. Biodistribution and lymphatic drainage of albumin.

Figure S13. Long-term biodistribution and lymphatic drainage of AL-SISIN-1.

Figure S14. Hemolysis test with AL-SISIN-1.

Figure S15. Histological assay with hematoxylin and eosin (H&E) staining of major organs.

Figure S16. Regression of tumor-draining lymph node enlargement by AL-SISIN-1.

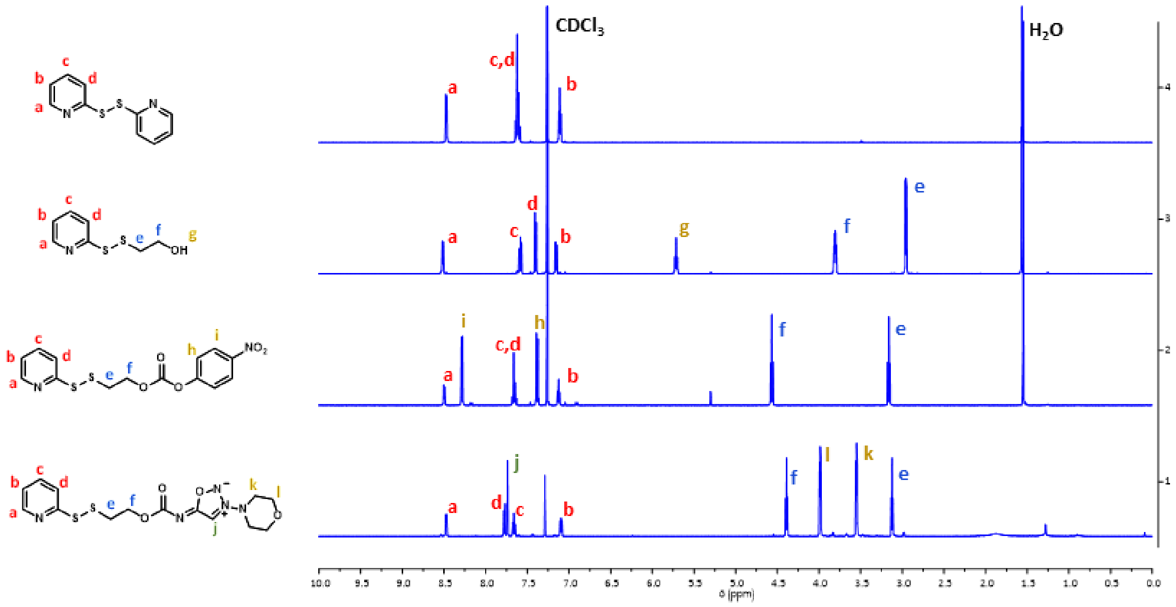

Figure S1. ^{1}**H NMR spectra of synthetic compounds including SISIN-1.**

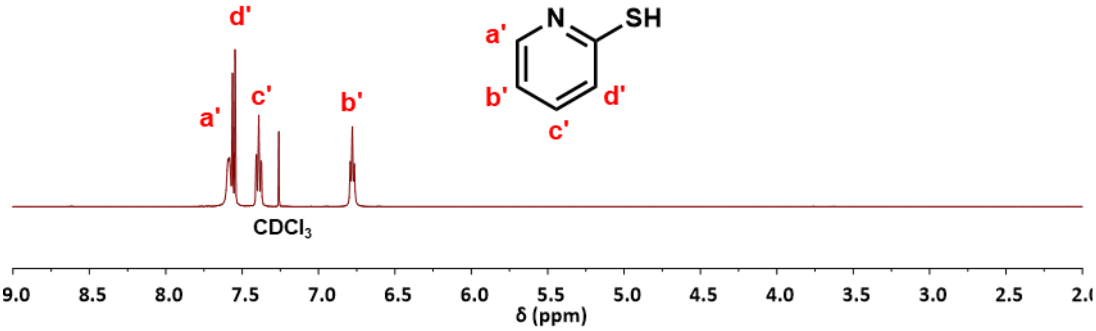

Figure S2. ^{1}**H NMR spectra of 2-mercaptopyridine.**

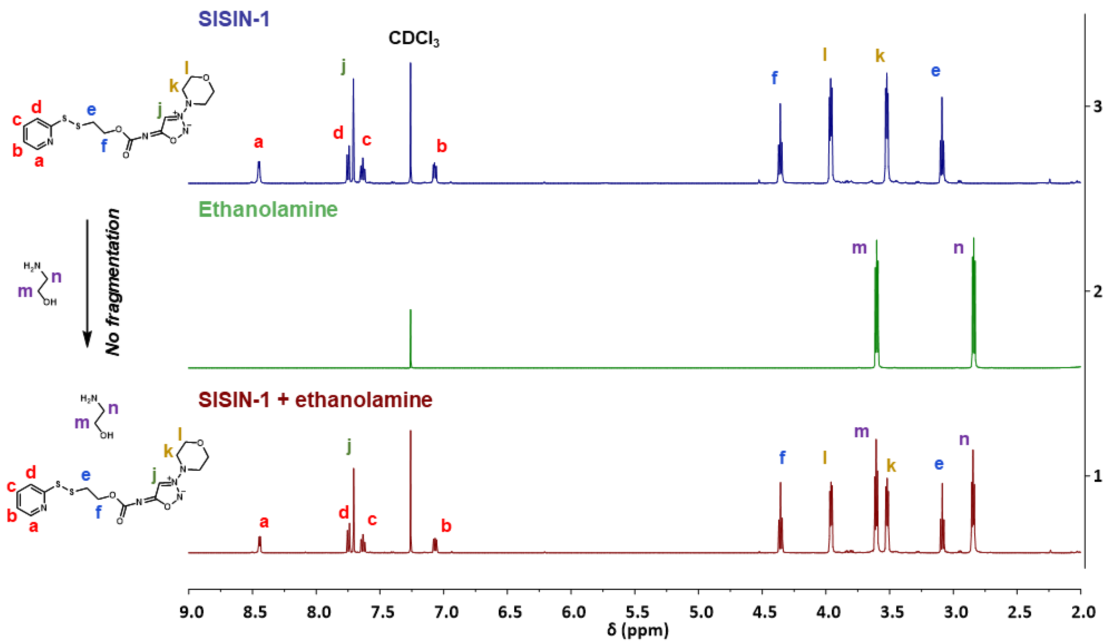

Figure S3. ¹H NMR spectra of SISIN-1 in the presence of a primary amine and a hydroxyl group. 2 equivalence of ethanolamine was treated into SISIN-1 in $CDCl_3$ and incubated at room temperature overnight. No characteristic peak change was found for both SISIN-1 and ethanolamine.

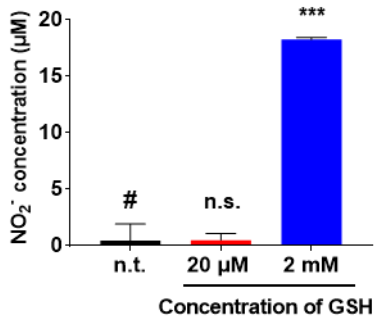

Figure S4. Redox-triggered NO release by SISIN-1. Quantification of NO release from SISIN-1 (50 μM). SISIN-1 was incubated under various physiological conditions at 37 °C for overnight. The amount of NO release was determined by Griess assay. Data are presented as mean ± SD (n=3), which were statistically analyzed with one-way ANOVA test by comparing # with the control groups. $***p < 0.001$; n.s. represents no significant difference.

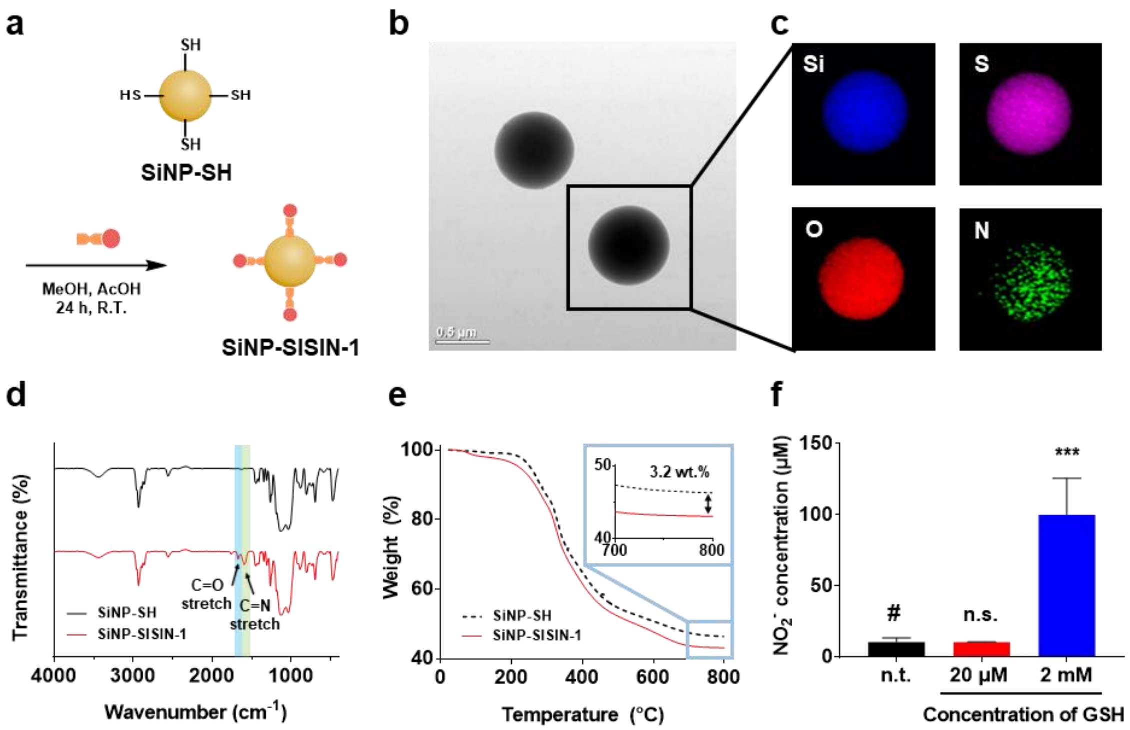

Figure S5. Characterization of SiNP-SISIN-1. (a) Preparation scheme of the SiNP-SISIN-1. (b) Representative TEM image of the SiNP-SISIN-1. (c) Corresponding energy-dispersive X-ray spectroscopy (EDS) mapping images of the SiNP-SISIN-1. Blue, pink, red, and green represents silicon, sulfur, oxygen, and nitrogen, respectively. (d) FT-IR spectra of SiNP-SH and SiNP-SISIN-1 in a KBr pellet. Each colored area indicates the characteristic bands of SISIN-1. (e) Thermogravimetric analysis (TGA) curves of SiNP-SH and SiNP-SISIN-1. 5 mg of each sample was loaded and heated from room temperature to 800 °C at a rate of 5 °C min^{-1} under N_2 atmosphere. Enlarged inset image represents the corresponding weight change at 800 °C. (f) Quantification of NO release from SiNP-SISIN-1 (2.5 mg mL^{-1}). SiNP-SISIN-1 was incubated under various physiological conditions at 37 °C in DMEM for overnight. The amount of NO release was determined by Griess assay. Data are presented as mean ± SD (n=6), which were statistically analyzed with one-way ANOVA test by comparing # with the control groups. ***$p < 0.001$; n.s. represents no significant difference.

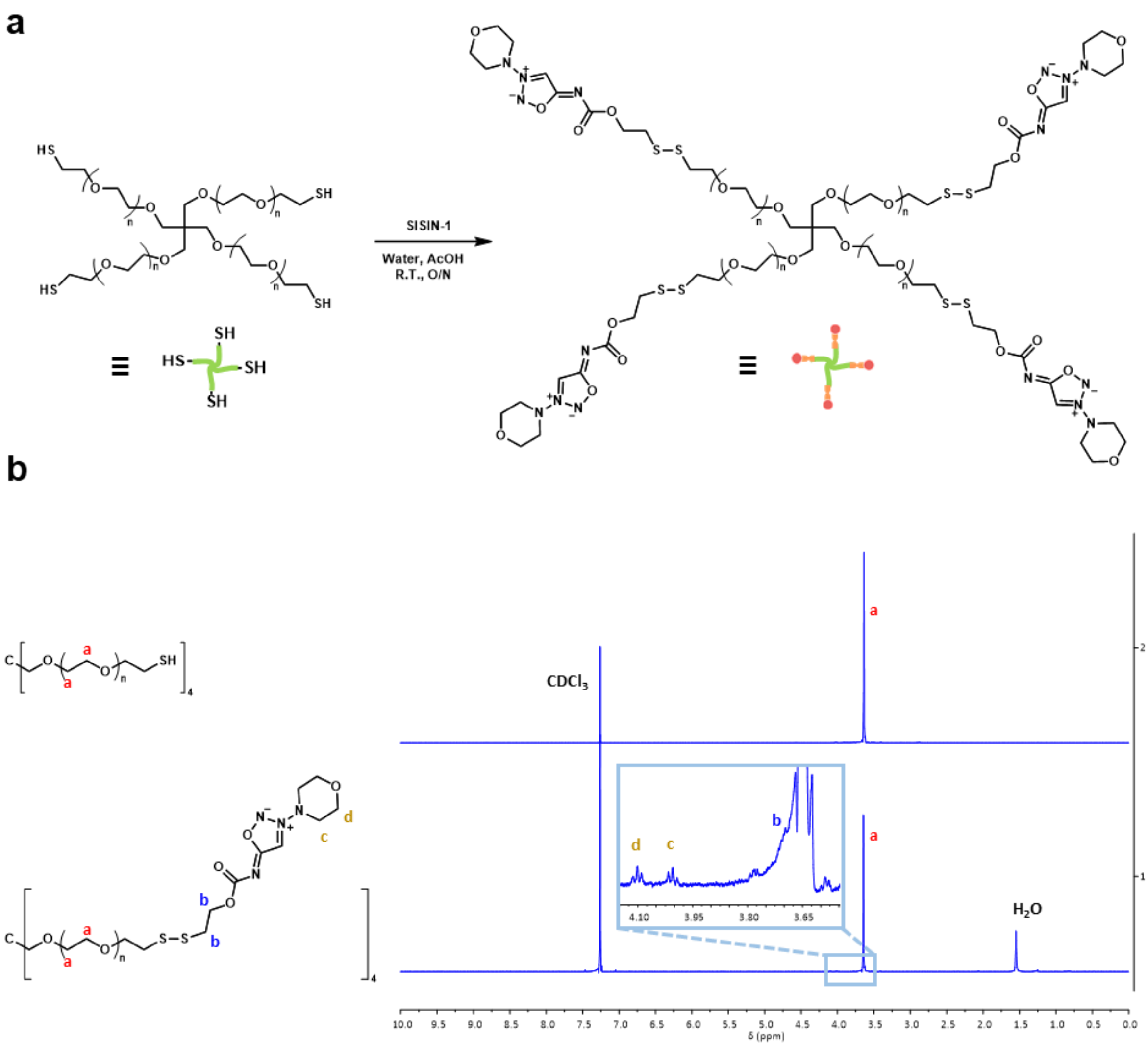

Figure S6. Preparation of 4-arm PEG-SISIN-1. (a) Synthetic route of the SISIN-1-conjugated 4-arm PEG (4-arm PEG-SISIN-1). (b) ^{1}H NMR spectra of synthetic compounds including 4-arm PEG-SISIN-1.

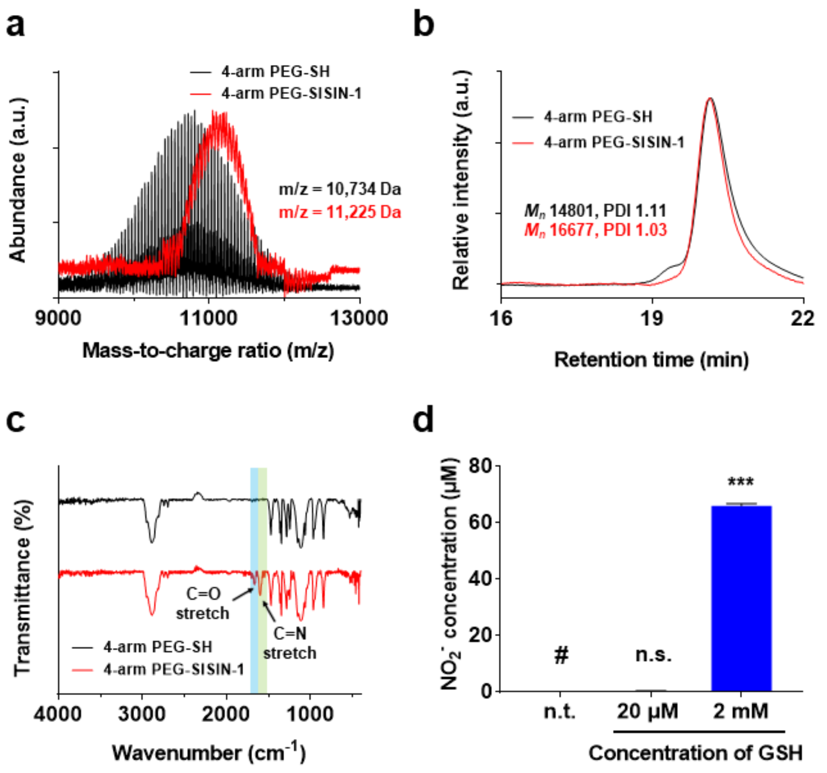

Figure S7. Characterization of 4-arm PEG-SISIN-1. (a) MALDI-TOF mass spectra of 4-arm PEG-SH and 4-arm PEG-SISIN-1. (b) GPC profile of 4-arm PEG-SH and 4-arm PEG-SISIN-1. The relative molecular weight was determined by comparing with polystyrene standards with THF as an eluent system. (c) FT-IR spectra of 4-arm PEG-SH and 4-arm PEG-SISIN-1 in a KBr pellet. Each colored area indicates the characteristic bands of SISIN-1. (d) Quantification of NO release by 4-arm PEG-SISIN-1 (50 µM). 4-arm PEG-SISIN-1 was incubated under various physiological conditions at 37 °C in DMEM for overnight. The amount of NO release was determined by Griess assay. Data are presented as mean ± SD (n=6), which were statistically analyzed with one-way ANOVA test by comparing # with the control groups. ****p* < 0.001; n.s. represents no significant difference.

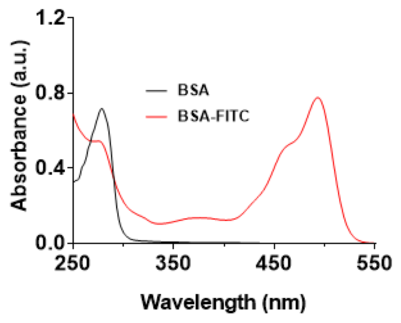

Figure S8. Characteristics of BSA-FITC. UV/Vis absorbance spectra of BSA (1 mg mL^{-1}) and BSA-FITC (0.1 mg mL^{-1}). Molar FITC to protein ratio (F/P) was determined to be 1.9, based on the manufacturer's protocol.

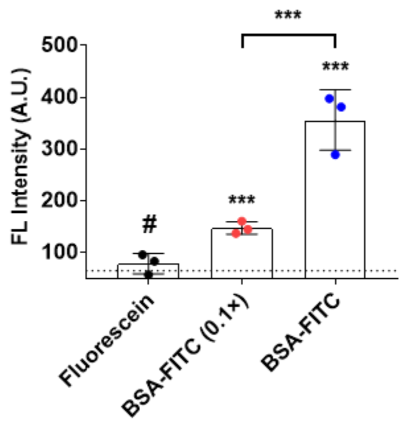

Figure S9. Size-dependent lymphatic drainage into lymph node. Each FITC-containing sample (fluorescein concentrations equivalent to 0.3 mM, 10 μL) was subcutaneously injected into the paw tissue of 8-week old female BALB/c mice. 2 h after the sample administration, draining lymph nodes were collected and homogenized for quantification. Fluorescence (FL) was determined with excitation/emission peak at 485/510 nm. Data are presented as mean ± SEM (n=3), which were statistically analyzed with one-way ANOVA test by comparing # with the control groups. ***$p < 0.001$. Dashed line indicates the FL intensity detected from DPBS-treated lymph nodes.

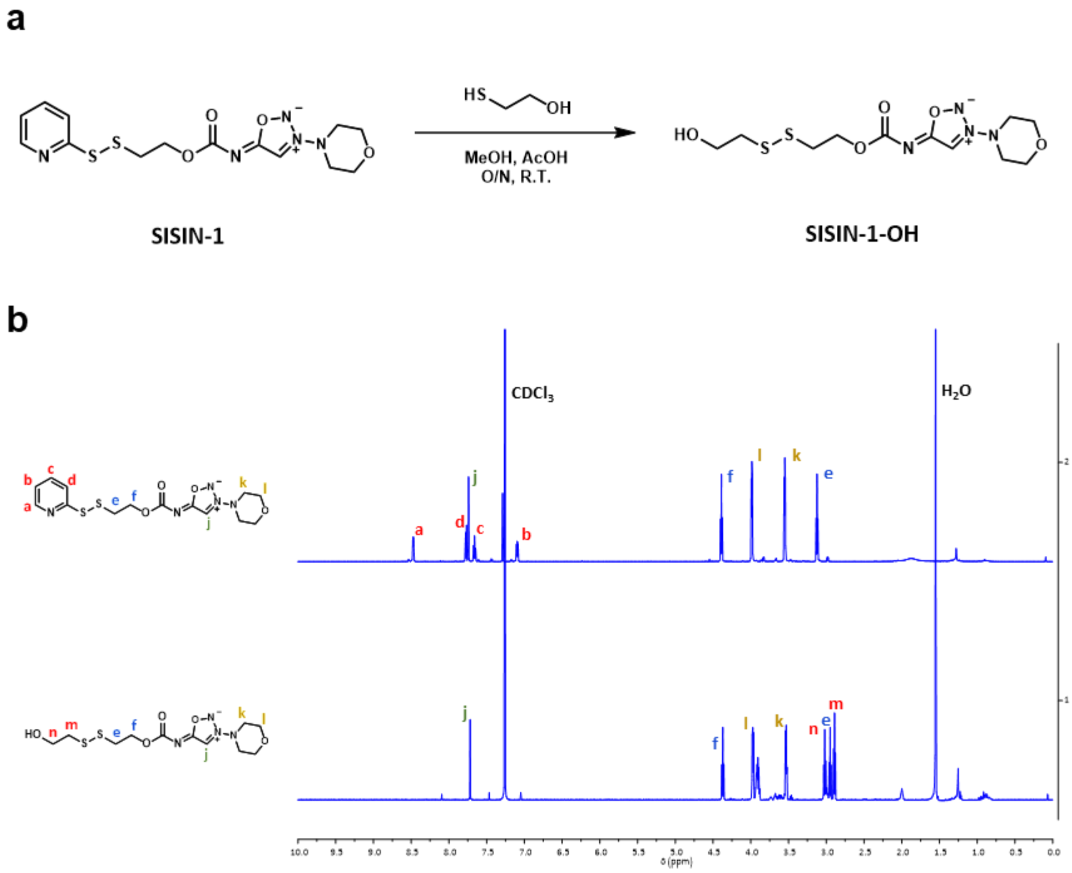

Figure S10. Preparation of self-immolative small molecule NO prodrug bearing a hydroxyl group. (a) Synthetic route of the alcohol-conjugated SISIN-1 (SISIN-1-OH). (b) ^{1}H NMR spectra of synthetic compounds including SISIN-1-OH.

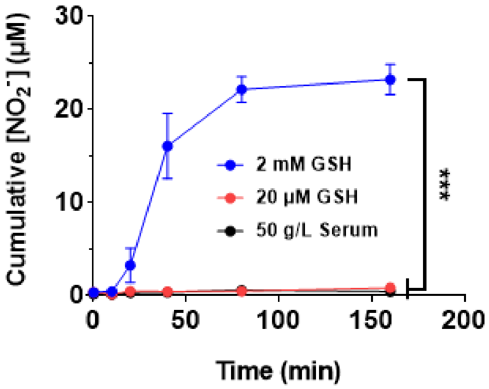

Figure S11. Cumulative NO release profile of SISIN-1-OH under reductive conditions. SISIN-1-OH (50 μM) was incubated under various physiological conditions at 37 °C in DMEM and the corresponding NO release was quantified by Griess assay. Data are presented as mean ± SD (n=4), which were statistically analyzed using two-way ANOVA. ****p* < 0.001.

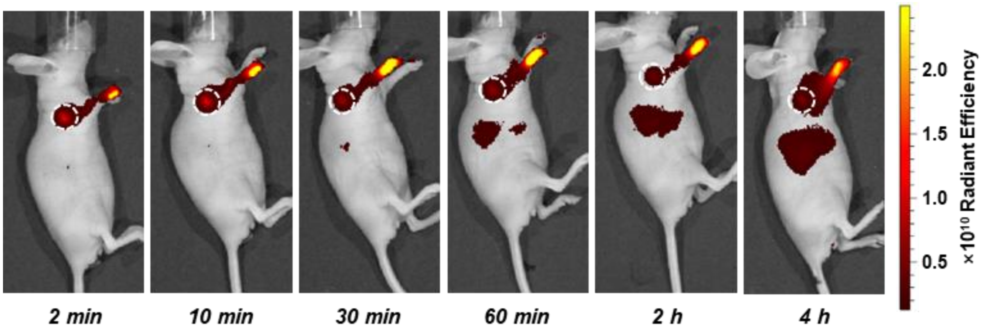

Figure S12. Biodistribution and lymphatic drainage of albumin. Time-dependent lymphatic accumulation and biodistribution of BSA-AF (2 mM, 10 μL) were visualized by intravital fluorescence imaging (excitation/emission peak at 640/710 nm). White circles represent the draining lymph nodes.

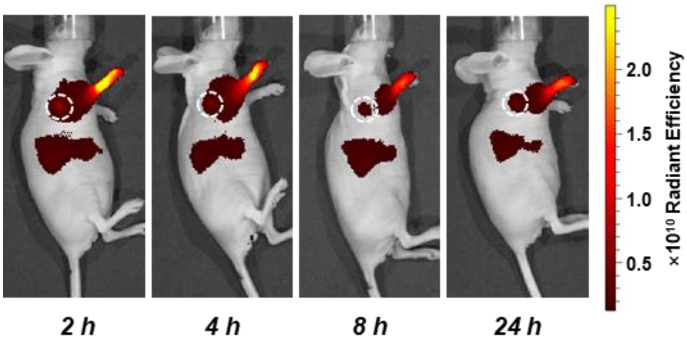

Figure S13. Long-term biodistribution and lymphatic drainage of AL-SISIN-1. Time-dependent lymphatic accumulation and biodistribution of AF647-AL-SISIN-1 (2 mM, 10 μL) were visualized by intravital fluorescence imaging (excitation/emission peak at 640/710 nm). White circles represent the draining lymph nodes.

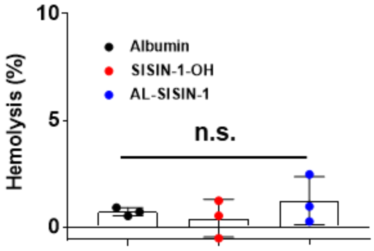

Figure S14. Hemolysis test with AL-SISIN-1. Data are presented as mean ± SEM (n=3), which were statistically analyzed with one-way ANOVA. n.s. represents no significant difference.

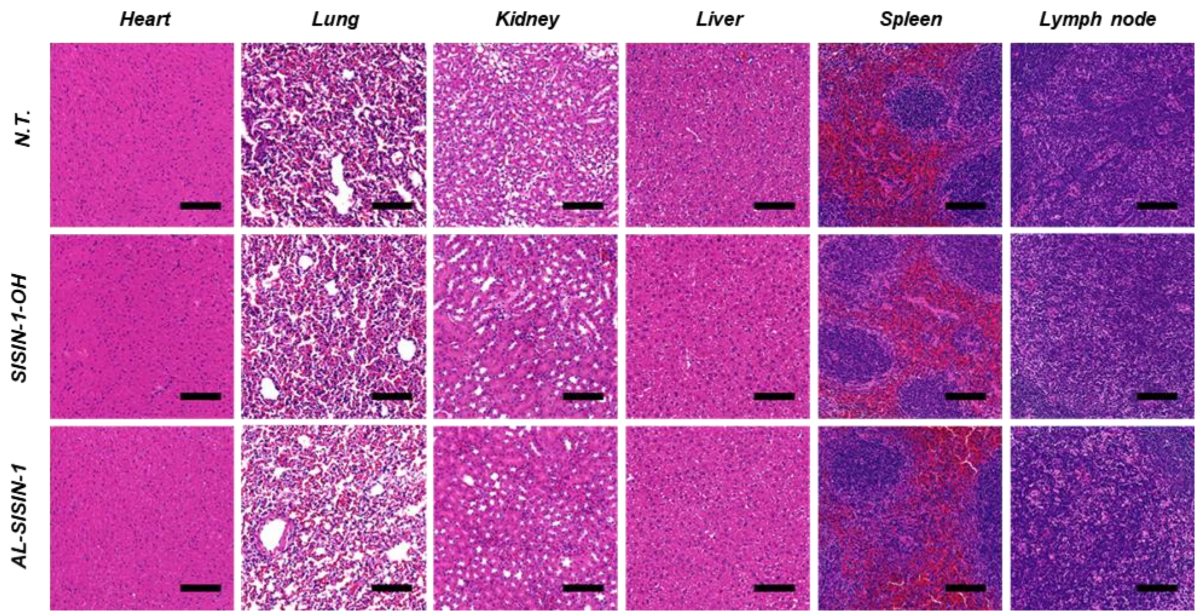

Figure S15. Histological assay with hematoxylin and eosin (H&E) staining of major organs. Histological sections of major organs of 8-week old female BALB/c were collected 3 days after the treatment of samples. Scale bar is 100 μm.

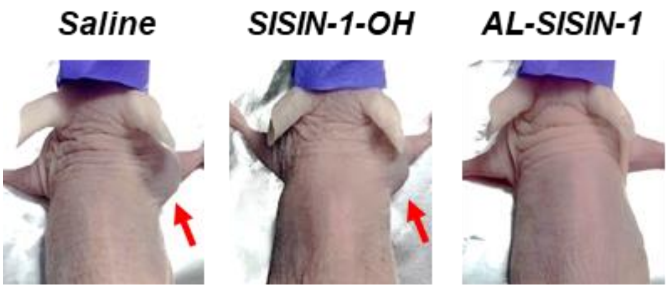

Figure S16. Regression of tumor-draining lymph node enlargement by AL-SISIN-1. Representative images of the sample-treated B16-F10 tumor-bearing BALB/c-*nu*/*nu* mice 14 days after the last sample injection. Red arrow shows the enlarged tumor-draining lymph node, indicating the presence of lymphatic metastasis.
